# Recurrent patterns after postoperative radiotherapy for early stage endometrial cancer: A competing risk analysis model

**DOI:** 10.1002/cam4.4423

**Published:** 2021-11-15

**Authors:** Kang Ren, Wenhui Wang, Shuai Sun, Xiaorong Hou, Ke Hu, Fuquan Zhang

**Affiliations:** ^1^ Department of Radiation Oncology Peking Union Medical College Hospital Chinese Academy of Medical Science & Peking Union Medical College Beijing China

**Keywords:** adjuvant radiotherapy, competing risk model, endometrial cancer, recurrence pattern

## Abstract

**Objective:**

The study aimed to evaluate site‐specific recurrent patterns via competing risks analysis and hazard function to provide evidence for adjuvant treatment and follow‐up for early staged endometrial cancer (EC).

**Methods:**

A total of 858 patients with International Federation of Gynecology and Obstetrics stage I–II EC who received adjuvant radiotherapy at our institution (2000–2017) were included. The radiotherapy modality comprised external beam radiotherapy (EBRT) with or without vaginal brachytherapy (VBT) or VBT alone. Competing risks analysis and hazard rate function were employed to evaluate the recurrence rate according to the ESMO–ESGO–ESTRO risk classification.

**Results:**

The 5‐year overall survival rates of the low‐risk (LR), intermediate‐risk (IR), high–intermediate risk (HIR), and high‐risk (HR) groups were 96.1%, 95%, 93%, and 89.7%, respectively (*p* = 0.018). Sixty‐eight patients developed recurrence. The 5‐year incidence of distant recurrence was the highest in the HR group (14.87%), followed by the HIR (7.71%), IR (5.27%), and LR (1.26%) groups (Gray's test, *p* < 0.001). The LR and IR groups showed late metastasis behaviors for distant metastasis. The HR group presented a large magnitude of distant metastasis with an early peak that increased beyond 3 years. Subgroup analysis revealed that EBRT±VBT tended to reduce the locoregional relapse rate compared with VBT in the HIR–HR group (2.36% vs. 7.73%, Gray's test, *p* = 0.08).

**Conclusion:**

The established competing risk modeling demonstrated different recurrence patterns across the risk groups and radiotherapy modes. A better understanding of the change in site‐specific recurrence behavior allows more targeted adjuvant treatment and surveillance regimens.

## Graphical abstract

The recurrence pattern of endometrial cancer is affected by risk groups and adjuvant treatment. The time‐varying competing risk analysis and hazard function modeling were established. The site‐specific recurrence patterns differed across the risk classification and adjuvant radiotherapy modality. More intense and targeted follow‐up schedules should be administrated to the high‐risk group.

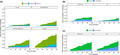

## 1 INTRODUCTION

Endometrial cancer (EC) has been one of the most common malignant gynecological tumors.^1^ Although around 75% of EC is diagnosed at an early International Federation of Gynecology and Obstetrics (FIGO) (2009 International Federation of Gynecology and Obstetrics) stage with a comparatively ideal prognosis, recurrence still occurs in patients treated with surgery and adjuvant therapy.^2^ Recurrent tumors lead to a significantly reduced survival rate, with the 5 ‐year overall survival (OS) decreasing to 55% for pelvic recurrences and 17% for extrapelvic recurrences.^2^ The overall recurrent rate for early stage EC is about 15%.^3^, ^4^ However, recurrent ECs have been reported to be heterogeneous representing diverse pathological types, tumor aggressiveness, and treatment responsiveness, especially in the early stage patient group.^5^ And the adjuvant treatment and follow‐up schemes for EC patients after adjuvant treatment are inconsistent across the guidelines.^6^, ^7^

Previous studies have shown that recurrence is influenced by several clinicopathologic factors, such as lymph‐vascular space invasion (LVSI), FIGO stage, depth of myometrial invasion (MMI), differentiation grade, and histology types.^5^, ^8^, ^9^, ^10^ Also, patients receiving different radiotherapy modalities also have shown different recurrence behavior.^5^, ^9^

Since early stage ECs have a relatively longer survival period and recurrence event varies considerably over time, evaluation of recurrence profiles is crucial to predict prognosis and guide surveillance.^11^ However, seldom studies investigated the site‐specific recurrence patterns over time. Recurrence patterns depicted by hazard function can reveal how risk changes over time and is affected by treatment interventions.^12^ Site‐specific recurrence profiles of different radiotherapy modalities according to European Society for Medical Oncology (ESMO)–European Society of Gynecological Oncology (ESGO)–European Society for Radiotherapy & Oncology (ESTRO) classification remain unclear, and there is a lack of homogenized cohort validation with similar adjuvant treatment regimen aimed at early stage ECs.

The present study aimed to evaluate the survival outcomes and clarify initial failure patterns by competing risk analysis and hazard rate function, thus providing useful prognostic evidence and helping to guide individualized targeted follow‐up schemes.

## 2 MATERIALS AND METHODS

### 2.1 Patients

Patients with 2009 FIGO stage I–II EC were analyzed between January 2000 and December 2017 at Peking Union Medical College Hospital. All enrolled patients completed post‐surgery adjuvant radiotherapy. Patients were excluded if they were previously diagnosed with another malignant tumor, did not complete adjuvant treatment, or had an insufficient follow‐up of less than 3 months and loss of clinicopathological data.

### 2.2 Treatment and follow‐up

All the patients were performed with preoperative clinical evaluation including pelvic and abdominal computed tomography (CT), magnetic resonance imaging (MRI), ultrasonography, bone scintigraphy, and positron emission tomography (PET) with the fluorodeoxyglucose (18F) to confirm the status of lymph node metastasis. Surgical treatment included a total hysterectomy and unilateral or bilateral salpingo‐oophorectomy with or without pelvic and para‐aortic lymph node dissection. Patients with confirmed negative preoperative lymph nodes and did not undergo lymph node staging would be classified into the cN0 group. Patients with confirmed lymph node metastasis would undergo lymphadenectomy or suspicious lymph node biopsy and would be categorized into the pN0 group after pathological confirmation of negative.

Adjuvant radiotherapy including external beam radiotherapy (EBRT), vaginal brachytherapy (VBT) alone, or a combination of both, was administered to all enrolled patients. EBRT was delivered to the pelvic area using a total dose of 45–50.4 Gy in 23–28 fractions with the intensity‐modulated radiotherapy (IMRT) technique, or three‐dimensional conformal radiotherapy (3D‐CRT) modality. High‐dose rate brachytherapy was delivered with a vaginal cylinder to the upper part of the vagina. Regimens included 5 Gy per fraction in five to six fractions prescribed to 5 mm below the vaginal surface in postoperative VBT alone. When VBT was used in combination with EBRT, doses of 5 Gy per fraction in two fractions were applied. The decision of chemotherapy depended on the surgeon's discretion, clinicopathologic conditions, and the willingness of the patients. The chemotherapy in our study mainly consisted of two regimens: (1) Weekly cisplatin for two to four cycles concurrent chemoradiotherapy during the EBRT followed by two cycles of intravenous carboplatin/paclitaxel at 21‐day intervals after radiotherapy. (2) Intravenous carboplatin/paclitaxel at 21‐day intervals for three to six cycles after surgery and followed by adjuvant radiotherapy.

Follow‐up was performed every 3 months for the first 2 years, every 6 months for the following 5 years, and once a year thereafter including physical examinations and imaging techniques.

### 2.3 Definition of risk classification

All of the enrolled patients were classified according to the ESMO–ESGO–ESTRO classification (2016 version) and divided into four classes: low risk (LR), intermediate risk (IR), high–intermediate risk (HIR), and high risk (HR).^13^

1. LR: Stage I endometrioid tumors, grade 1–2, <50% MMI, LVSI negative;
2. IR: Stage I endometrioid tumors, grade 1–2, ≥50% MMI, LVSI negative;
3. HIR: Stage I endometrioid tumors, grade 3, <50% MMI, regardless of LVSI status or Stage I endometrioid tumors, grade 1–2, LVSI unequivocally positive, regardless of MMI; and
4. HR: Stage I endometrioid tumors, grade 3, ≥50% MMI, regardless of LVSI status, Stage II, non‐endometrioid (serous or clear‐cell or undifferentiated carcinoma, or carcinosarcoma).

For subgroup analysis, LR and IR were merged to form the “LR–IR” group and the HIR and HR were merged to form the “HIR–HR” group.

### 2.4 Survival outcome and recurrence events

The primary endpoints of the study were OS, defined as the time from primary surgery to death caused by any reason. Tumor recurrence, defined as the time from primary surgery to the date of recurrence, was classed as locoregional (vaginal or regional) recurrence and distant metastasis. Local‐regional failure‐free survival (LRFS) and distant metastasis failure‐free survival (DMFS) were applied in subgroup analysis. LRFS was calculated from the date of surgery to the date of vaginal stump recurrence or regional lymphatic drainage area failure or death due to any cause. DMFS was calculated from the date of surgery to the date of distant metastasis failure or death due to any cause or the last follow‐up time.

Initial recurrence events were documented hierarchically according to the worst site as follows: (1) vaginal recurrence, defined as isolated recurrence within vaginal walls or vaginal cuff; (2) regional recurrence, defined as intrapelvic sites, pelvic lymph nodes, or regional lymph nodes including the retroperitoneal nodes metastasis; and (3) distant recurrence, defined as any recurrence involving a remote organ site (e.g., lung, liver, brain, bone, and sub‐diaphragmatic lymph nodes).

Recurrences were assessed by physical examination and imaging techniques: CT, MRI, ultrasonography, bone scintigraphy, and PET/CT as well as histological findings if available.

### 2.5 Statistical analysis

The distribution of clinicopathologic characteristics was compared between risk groups using the Kendall's tau‐c test. The Kaplan–Meier and the log‐rank test were applied to estimate and compare the OS and distributions using SPSS (IBM).

The cumulative risk incidence of recurrence pattern was evaluated by competing risk analyses (Gray's test), treating initial recurrences in nontarget‐type sites, and death without recurrence as competing events. Competing risks regressions were applied to estimate sub‐hazards ratios and 95% confidence interval (CI) adjusted for other factors such as age and chemotherapy to evaluate the associations between the risk of a specific recurrence site and the EC subclassification and radiotherapy modality. The competing risk analysis and *p*‐values were calculated by the Wald test using the cmprsk package with R version 3.0.1 (R Project for Statistical Computing, Vienna, Austria).

The cumulative incidence curves were performed using the survminer package of R software (https://rpkgs.datanovia.com/survminer/index.html). The colored stacks represented state occupation probabilities of the first failure and death events according to each risk group and radiotherapy mode.

The hazard rate function was used to estimate the first recurrence pattern using the time by kernel smoothing method. *p*‐values were derived using two‐sided tests and values <0.05 were considered statistically significant.

## 3 RESULTS

### 3.1 Characteristics of the study population

Of the 858 patients included, LR, IR, HIR, and HR accounted for 35.1%, 29.4%, 19.3%, and 16.2%, respectively. Among the study population, 312 (36.4%) patients received EBRT with or without VBT and 546 (63.6%) patients received VBT alone. A total of 312 patients received EBRT. Among them, the number of patients who received 3DCRT and IMRT was 122 and 190, respectively. For the EBRT group, the range of cumulative EQD2 using the linear‐quadratic model, with alpha/beta ratios of 10 for target was 44.25–50 Gy. For the VBT alone group, the cumulative EQD2 was 31.25–37.5 Gy. For the EBRT+VBT group, the cumulative EQD2 was 56.75–62.5 Gy.

### 3.2 Comparison by risk groups

VBT was more commonly administrated in the LR and IR groups than in the HIR and HR groups, while patients in the HR group received EBRT±VBT more frequently (*p* < 0.001). The distribution of MMI, FIGO stage, and histological type differed significantly across the risk classification and radiotherapy modes (*p* < 0.001). Additionally, adjuvant chemotherapy was administrated more frequently in HR and HIR groups (*p* < 0.001). Of note, recurrence was more common in the HR and HIR groups, and the HR group had a higher rate of distant metastasis and regional recurrence than the other three groups (*p* < 0.05) (Table 1).

**TABLE 1:** Main characteristics of the patients

	Risk classifications				
	LR (*n* = 301)	IR (*n* = 250)	HIR (*n* = 164)	HR (*n* = 143)	*p* *
Age (years)					
<60	213 (70.8%)	135(54.0%)	115 (70.5%)	94 (65.7%)	0.413
≥60	88 (29.2%)	115(46.0%)	49 (29.5%)	49 (35. 1%)	
MMI					
<50%	299 (99.3%)	4 (1.6%)	122 (74.4%)	57 (39.9%)	<0.001
≥50%	2 (0.7%)	246 (98.4%)	42 (26.5%)	86 (60.1%)	
LVSI					
Negative	301 (100%)	250 (100%)	61 (37.2%)	98 (68.5%)	<0.001
Positive	0 (%)	0 (%)	103 (62.8%)	45 (31.5%)	
FIGO stage					
Ⅰa	299 (99.3%)	7 (2.8%)	119 (72.6%)	24 (16.8%)	
Ⅰb	2 (0.7%)	243 (97.2%)	45 (27.4%)	57 (39.9%)	**0.001**
Ⅱ	0	0	0	62 (43.4%)	
Histological types					
Type I	300 (99.7%)	248 (99.2%)	162 (98.8%)	100 (69.9%)	<0.001
Type II	1 (0.3%)	2 (0.8%)	2 (1.2%)	43 (30.1%)	
Mode of surgery					
Open approaches	10 (33.4%)	80 (32%)	47 (28.7%)	47 (32.9%)	0.367
MIS	135 (44.9%)	115 (46%)	101 (61.6%)	62 (43.4%)	
Lymphadenectomy					
No	100 (33.2%)	96 (38.1%)	30 (18.1%)	38 (27.3%)	<0.001
Yes	201 (66.8%)	156 (61.9%)	136 (81.9%)	101 (72.7%)	
Radiotherapy modes					
EBRT±VBT	68 (22.6%)	90 (36%)	49 (29.9%)	105 (73.4%)	<0.001
VBT	233 (77.4%)	160 (64%)	115 (70.1%)	38 (26.6%)	
Adjuvant chemotherapy					
Yes	9 (3.0%)	13 (5.2%)	25 (15.2%)	59 (41.3%)	<0.001
No	292 (97%)	237(94.8%)	139 (84.8%)	84 (58.7%)	
Adjuvant treatment					
EBRT±VBT+chemo	4 (1.3%)	13 (5.2%)	12 (7.3%)	48 (33.6%)	<0.001
VBT+chemo	5 (1.7%)	0	16 (9.8%)	11 (7.7%)	
First recurrent patterns					
Non‐recurrence	288 (95.70%)	234 (93.6%)	148 (90.2%)	120 (83.9%)	**<0.001**
Vaginal	2 (0.7%)	2 (0.8%)	2 (1.2%)	1 (0.7%)	
Regional	4 (1.3%)	4 (1.6%)	4 (2.4%)	5 (3.5%)	
Distant	7 (2.3%)	10 (6.1%)	10 (6.1%)	17 (11.9%)	

Bold indicates statistically significant *p*‐value <0.05. Abbreviations: chemo, chemotherapy; FIGO, International Federation of Gynecology and Obstetrics; HIR, high–intermediate risk; HR, high risk; IR, intermediate risk; LR, low risk; LVSI, lymph‐vascular space invasion; MIS, minimally invasive surgery; MMI, myometrial invasion.

* *p* are based on the Kendall's tau‐c test.

### 3.3 Survival outcomes in different subgroups

**FIGURE 1:**
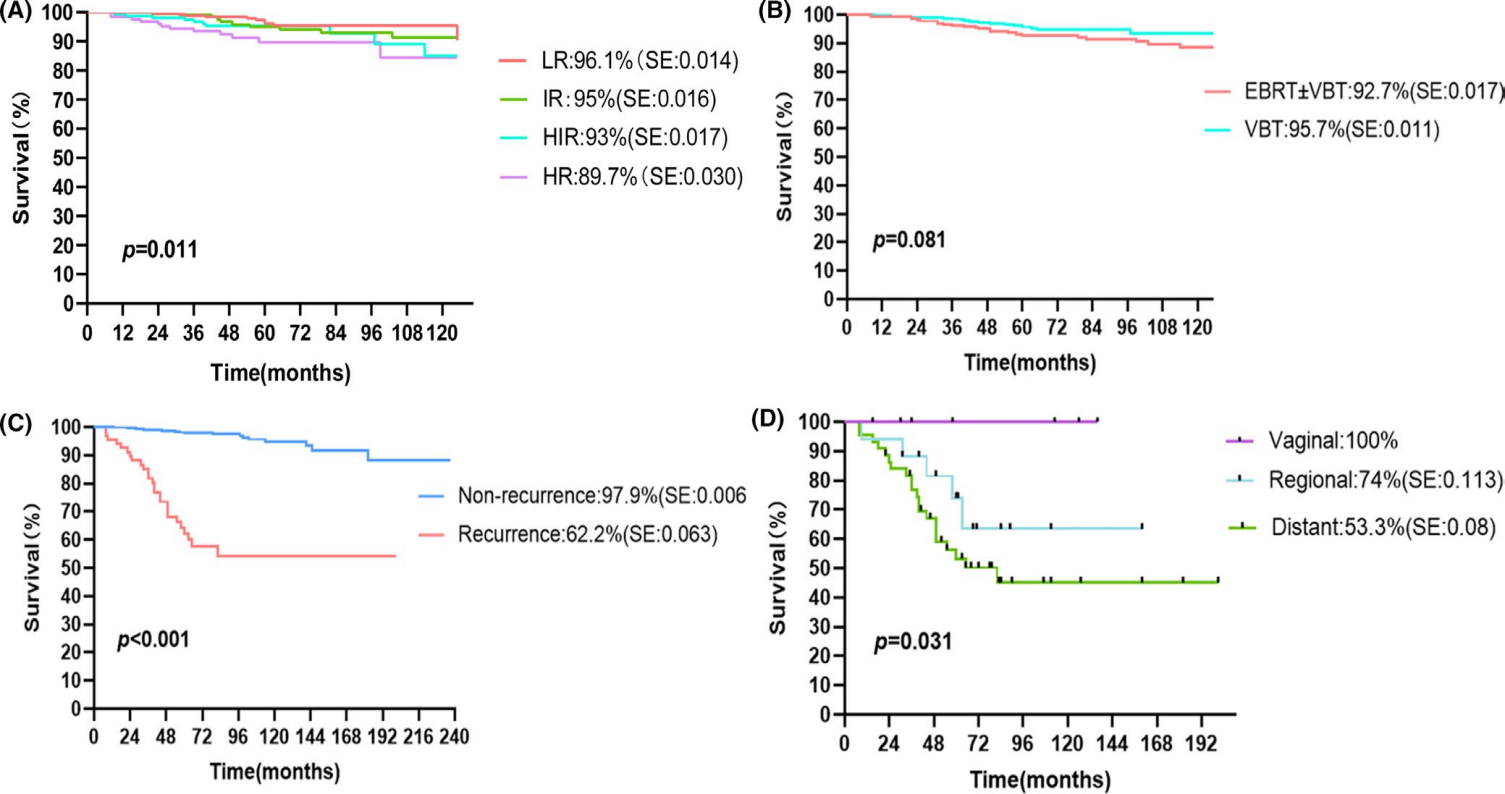
Kaplan–Meier survival curves: (A) the 5‐year overall survival stratified by risk groups; (B) the 5‐year overall survival stratified by radiotherapy modalities; (C) patients with and without recurrence; and (D) patients with different recurrent patterns

The median follow‐up period was 59 months (range, 3–237 months). The 5‐year OS rates of LR, IR, HIR, and HR were 96.1%, 95%, 93%, and 89.7%, respectively (*p* = 0.011). In the whole group, 5‐year OS of the patients who received EBRT±VBT and VBT was 92.7% (95% CI 89.4%–96%) and 95.7% (95% CI 93.5%–97.8%), respectively (*p* = 0.081) (Figure 1B). In the LR–IR group, the 5‐year OS of patients who received EBRT±VBT and VBT was 92.9% (95% CI 88.4%–97.4%) and 96.7% (95% CI 94.4%–99.1%), respectively (*p* = 0.066). In the HIR–HR group, the 5‐year OS was 92.6% (95% CI 88%–96.4%) and 93.2% (95% CI 88.9%–97.5%), respectively (*p* = 0.999). Comparisons between the two radiotherapy modes in more subgroups are presented in Table S3.

The 5‐year OS of patients with recurrence decreased to only 62.2% (95% CI, 49.9%–74.5%), whereas patients without recurrence had a significantly higher 5‐year OS of 97.9% (95% CI 96.7%–99.0%) (*p* < 0.001) (Figure 1C). The 5‐year OS of patients with vaginal, regional, and distant recurrences were 100%, 74%, and 53.3%, respectively (95% CI 37.6%–69.1%) (*p* = 0.031) (Figure 1D).

### 3.4 Recurrence patterns

The median time to recurrence of patients with relapse was 26 months (range 6–138). During follow‐up, 7.9% (68/858) of patients experienced recurrence. Among patients with recurrence, 10.3% (7/68) of patients had vaginal recurrences, 25% (17/68) of patients had regional relapses, and 64.7% (44/68) of patients had distant metastasis. The proportion of distant failure was higher than locoregional relapse across the four risk groups (53.8%, 62.50%, 62.50%, and 73.90% for LR, IR, HIR, and HR, respectively). The multistate plots intuitively visualized that the risk of distant metastasis increased from the LR to HR group (Figure 3A).

Table 2 shows the 5‐year cumulative incidence rates of site‐specific events based on the univariate analysis competing risks analysis. Table S1 shows the subdistribution hazard rates and their 95% CI values from the competing risks regression model, adjusting for age and chemotherapy received.

**TABLE 2:** Competing risks analysis for 5‐year cumulative rates of recurrence according to risk groups

Site of first recurrence	LR	IR	HIR	HR	*p* *
	*n* = 301	*n* = 250	*n* = 164	*n* = 143	
Vaginal	0.72 (0.72, 0.73)	0.45 (0.45, 0.46)	1.64 (1.61, 1.67)	0.86 (0.85, 0.88)	0.84
Regional	1.67 (1.66, 1.68)	2.12 (2.10, 2.15)	2.50 (2.47, 2.53)	5.11 (5.00, 5.21)	0.37
Distance	1.26 (1.25, 1.27)	5.27 (5.22, 5.32)	7.71 (7.59, 7.83)	14.78 (14.55, 15.02)	<0.001

Abbreviations: HIR, high–intermediate risk; HR, high‐risk; IR, intermediate‐risk; LR, low‐risk.

* Gray's test.

No significant distribution was observed for the vaginal and regional recurrence across the risk groups (*p* > 0.05). The 5‐year cumulative incidence of distant recurrence was highest in the HR group (14.78%) followed by the HIR (7.71%), IR (5.27%), and LR (1.26%) groups (Gray's test, *p* < 0.001). In competing risks regression analysis, hazard rates of distant metastasis were 3.78 (95% CI 1.033–13.84) for the IR group, 6.02 (95% CI 1.672–21.67) for the HIR group, and 12.34 (95% CI 3.454–24.11) for HIR group, relative to the LR group (*p* < 0.05) (Table S1).

Subgroup analysis to compare the recurrence rate in the EBRT±VBT and VBT groups showed no significant difference or trend for distant metastasis, irrespective of the LR–IR or HIR–HR group (*p* > 0.05). Notably, the locoregional rate for the EBRT±VBT and VBT groups in the LR–IR group was 2.90% and 2.46%, respectively. And the hazard rate of patients who received VBT was 0.72, relative to EBRT±VBT (*p* = 0.61). However, patients in the group of HIR–HR who received VBT alone revealed a trend for a higher locoregional recurrence rate compared with EBRT±VBT (7.73% vs. 2.36%, *p* = 0.08) (Table 3). The competing risks regression analysis showed the hazard rate of locoregional rate in the VBT group was 3.06 (95% CI 0.93–11.30) relative to the EBRT±VBT group (*p* = 0.08) (Table S2).

**TABLE 3:** Competing risks analysis for 5‐year cumulative rates of recurrence according to radiotherapy modality in sub‐risk group

	Site of first recurrence	EBRT±VBT	VBT	*p* *
LR–IR	Locoregional	2.90 (2.86, 2.94)	2.46 (2.44, 2.48)	0.60
	Distance	3.76 (3.71, 3.82)	2.80 (2.78, 2.82)	0.51
HIR–HR	Locoregional	2.36 (2.32, 2.39)	7.73 (7.59, 7.87)	0.08
	Distance	13.13 (12.94, 13.33)	8.02 (7.91, 8.13)	0.30

Abbreviations: HIR, high–intermediate risk; HR, high‐risk; IR, intermediate‐risk; LR, low‐risk.

* Gray's test.

### 3.5 Time‐varying recurrence profiles according to risk classes and radiotherapy modes

**FIGURE 2:**
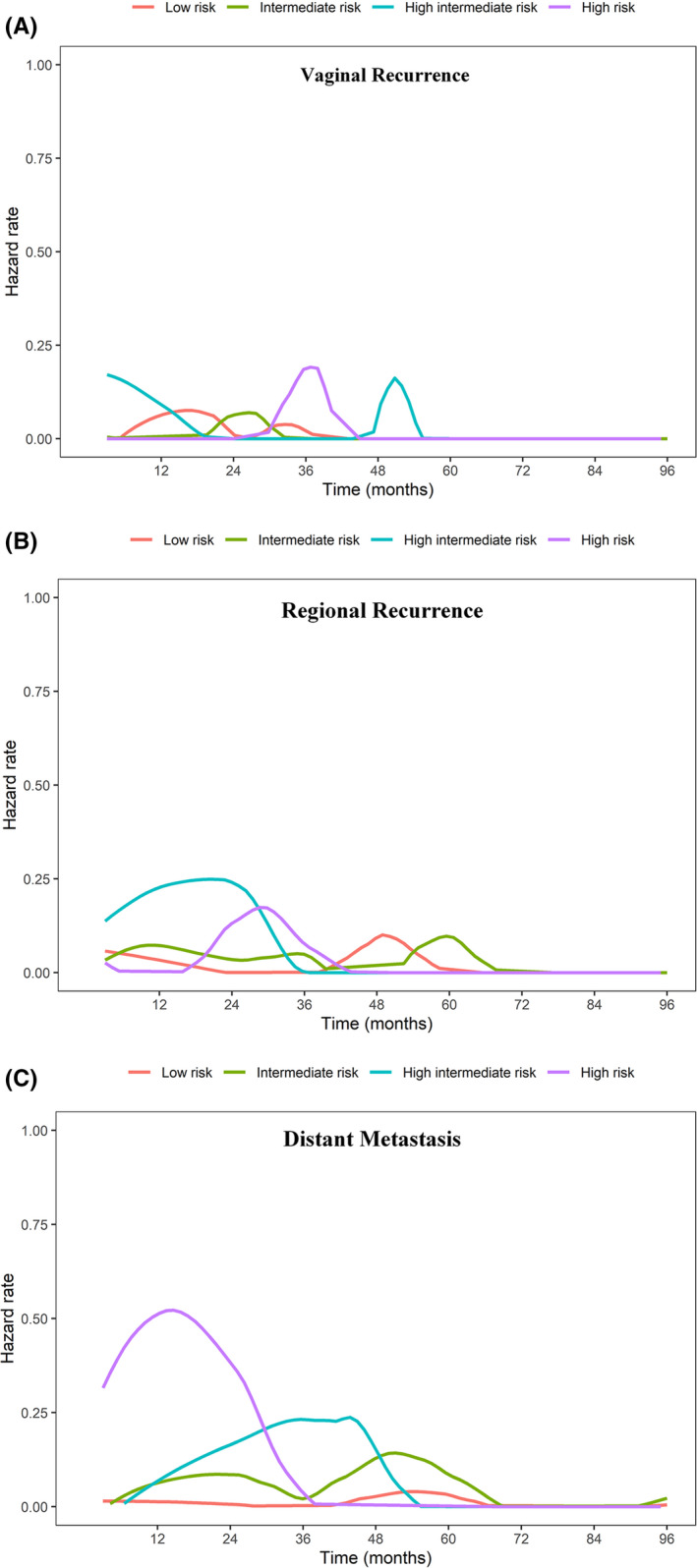
Hazard function plots representing failure sites over follow‐up time according to the risk groups: (A) vaginal recurrence; (B) regional recurrence; and (C) distant metastasis

Figure 2 represents the hazard function of the first recurrence sites. For vaginal recurrence, the HIR and HR groups indicated higher hazard rates and their peaks of relapse lagged behind the LR and IR groups (Figure 2A). For regional relapse, the HIR group presented the earliest peak of recurrence at around the second year after treatment, followed by the HR group peaking at 2.5 years, whereas the LR and IR groups showed delayed peaks of recurrence, which noted at the fourth year and fifth year, respectively (Figure 2B). As for distant failure, the HR group showed a higher magnitude of recurrence peak than the other three groups, which early increased from the sixth month, reached the maximum peak at the first year, and decreased to a negligible level after 3 years. HIR group revealed a lower hazard rate than the HR group and its peak of recurrence appeared after the third year. It was noteworthy that the LR and IR groups presented late metastasis behaviors with the peak of recurrence appearing at nearly the fifth year after treatment (Figure 2C).

**FIGURE 3:**
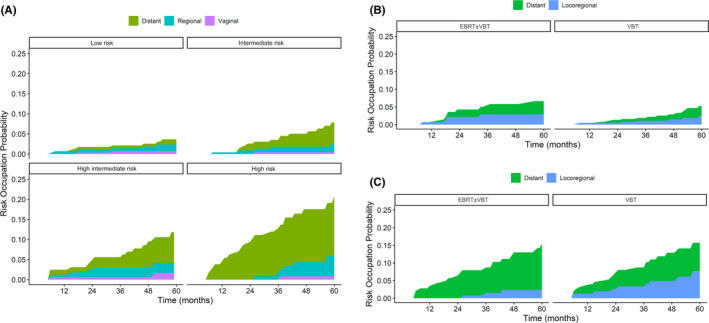
Multistate plots for risk occupation probabilities: (A) multistate based on the risk groups; (B) multistate based on the radiotherapy modes in the LR–IR groups; and (C) multistate based on the radiotherapy modes in the HIR–HR groups. HIR, high–intermediate risk; HR, high‐risk; IR, intermediate‐risk; LR, low‐risk

In the subgroup analysis of the LR–IR group, patients who received VBT alone showed a similar hazard rate of locoregional relapse with the EBRT±VBT group, except with a different shape and peak timing of recurrence (Figure S1A). However, in the HIR–HR group of patients with locoregional recurrence, the VBT group showed a higher magnitude of recurrence peaks than the EBRT±VBT and displayed an initial surge at the second year and a second small peak of recurrence 4 years after treatment (Figure S1C). Figure 3C also indicated that the VBT group presented a larger probability of locoregional recurrence than the EBRT±VBT group, but this trend was not observed in the LR–IR group.

## 4 DISCUSSION

The present study innovatively applied the time‐varying competing risk model and hazard function to visualize the first recurrence profiles for early stage ECs. The results confirmed that main failure patterns significantly differed across the risk groups and the radiotherapy modalities. Furthermore, EBRT±VBT showed an obvious trend of a lower rate of locoregional recurrence than VBT alone in the HIR–HR group. Of importance, the recurrence dynamics depicted by hazard function indicated different biological behavior of metastases, which may provide useful information for guiding follow‐up recommendations and for the management of adjuvant treatment.

The strengths of our study lie in that it included a relatively large cohort of early stage ECs who received adjuvant radiotherapy with a median follow‐up of 59 months, comprehensive clinicopathologic parameters were performed and compared. Our results showed that recurrence significantly impaired survival. Vaginal‐only recurrence showed the longest survival period without recurrence‐related death, while distant metastasis markedly impaired survival, with the 5‐year OS decreasing to 53.3%. The competing risk model was employed to mitigate the estimation bias and further investigate the initial three recurrence failure patterns. Compared with the conventional Kaplan–Meier or Cox survival analysis on the recurrence patterns, which can only deal with one type of event independently of its cause, competing risk regression can correct for differences in intercurrent death and censoring because of clinical necessities to estimate and compare the cumulative event‐free time.^9^, ^14^ Furthermore, the hazard rate function can provide variable‐over‐time information about the recurrence course to help make inferences on the metastasis biology and adjust the adjuvant treatment and surveillance regimens.^15^, ^16^

The overall recurrence rate was 7.9%, which was consistent with those reported in previous studies.^3^, ^8^, ^17^ The 5‐year OS of the whole population was 94.6% and declined in the order LR (96.1%), IR (95%), HIR (93%), and HR (89.7%), the results of survival rate were slightly higher than that reported by Bendifallah et al., the 5‐year survival rates were 80.7% for the whole population, 89.0% for LR, 91.7% for IR, 83.2% for HIR, and 67.9% for HR group.^8^ This discrepancy may due to the heterogeneity of patients, the proportion of HR patients was higher than our study (43% vs. 16.6%) and only 51% of patients received radiotherapy in their study. And adjuvant radiotherapy which was administrated to all enrolled patients in the present study improved the survival outcomes to some degree.^3^, ^8^, ^18^, ^19^ The present study corroborated that distant metastasis was more common than locoregional relapse across the four risk classifications for patients who received adjuvant radiation, but the trend was not obvious in the LR group.^3^ The proportion of distant failure was 53.8%, 62.50%, 62.50%, and 73.90% in the order of LR, IR, HIR, and HR groups, which were comparable to studies from Vizza et al. and Creutzberg et al.^4^, ^20^ E. Vizza et al. enrolled 71.6% of patients who received radiotherapy and found that distant failure was more common than locoregional recurrence (64% vs. 36%). The proportion of distant failure in the LR, IR, HIR, and HR groups was 43.75%, 100%, 75%, and 65.2%, respectively.^20^ In contrast to the recurrence patterns reported in our study, the most common recurrence sites were vagina or regional areas in the group of patients who did not receive adjuvant radiotherapy.^3^, ^8^ For example, the proportions of distant failure in the study of Bendifallah et al. were 27.3%, 37.5%, 33.3%, and 45.6% for LR, IR, HIR, and HR groups, respectively. Only 51% of patients in their study received radiotherapy.^8^ Likewise, Creutzberg et al. revealed that vaginal recurrences were the most common recurrences in the patients without radiotherapy, while more relapses occurred at distant sites for patients who received adjuvant radiotherapy.^4^ In addition, competing risks analysis demonstrated that risk classification did not affect vaginal or regional recurrence, since radiotherapy improved the locoregional rate and weakened correlation between risk groups and locoregional recurrence risks.^21^ Notably, 5‐year cumulative distant failure rates gradually increased from the LR to HR group (1.26%, 5.27%, 7.71%, and 14.8%), which were similar to the result of Bendifallah et al.^8^ HR group showed the highest rate of distant metastases and earlier metastasis behavior, highlighting that a novel adjuvant treatment regimen is needed, such as advancing the timing of adjuvant treatment and administration of the system chemotherapy for this group of patients.

An important highlight in our study is the construction of the subtype‐dependent and time‐varying modeling of recurrence. Our results verified that most relapses occurred within 3 years after treatment, which has been confirmed by several studies.^6^ Furthermore, we found the multiple‐peak pattern of recurrence, which differed across the risk groups over time. The closest related work to our study is that of Bendifallah et al. and Ignatov et al., both studies have explored the recurrence patterns according to the risk groups.^8^, ^22^ Sofiane Bendifallah et al. demonstrated that the rate of distant relapse was the highest in the HR group (20.7%) and the rate of locoregional recurrence was higher in the group of HR and HIR (24.3% and 16.6%, respectively) than in the group of LR and IR (6.5% and 6.6%, respectively).^8^ These results are comparable with the present study. In addition, this study indicated the different peaks of recurrence for different risk groups, that patients in the HR group presented the highest hazard of distant recurrence within the third year after treatment, whereas the “non‐high‐risk” groups (HIR, IR, and LR) showed delayed peaks of recurrence that appeared beyond 3 years after treatment. The interesting phenomenon of delayed relapse was also observed in lung and breast cancers.^23^, ^24^ However, plausible mechanisms for the late recurrence behaviors have not been clarified. Several explanations have been proposed to explain this phenomenon in breast cancer and lung cancer, such as tumor homeostasis, tumor dormancy, and treatment‐related enhancement of metastatic.^16^, ^23^ We hypothesize that micrometastases in the unirradiated areas proliferated again and developed recurrence events after a period of tumor dormancy. The effect seems to be more pronounced in the group of “non‐high‐risk” patients, probably due to a longer period of tumor accumulation. Besides, it should be noted that 10%–15% of high–intermediate patients harbor a p53 mutant that is considered an independent prognostic factor for distant recurrence.^25^, ^26^, ^27^ Thus, wide heterogeneity within the same risk group may lead to late metastatic behavior, highlighting the need to differentiate the risk stratification by adding new biomarkers.^27^, ^28^

Different from the related studies, which only reported the timing of the overall recurrence, our study further visually illustrated the risk‐based and site‐specific recurrence profiles via hazard rate function and multistate plots, which may provide more significant information on the surveillance. The routine follow‐up schemes comprised physical examinations, such as gynecological examinations and imaging.^29^ However, no randomized or prospective studies have evaluated the proper time interval and screening tools personally. The overall vaginal recurrence risk remained low, but a late increase in hazard rate was observed in the HIR and HR groups with the peak time appearing at 3 and 4.1 years, respectively, suggesting that gynecological examination should not be dispensable at each time follow‐up even after 3 years for HIR–HR patients. Furthermore, considerable attention should be paid to delay vaginal bleeding. The patients in the HR group developed early metastasis behaviors and survived the highest levels of distant metastasis rate within the 3 years after the end of treatment, highlighting that an intense follow‐up is needed for the HR group during this period. Furthermore, late metastasis behaviors were observed in “non‐high‐risk” groups (LR, IR, and HIR), a prolonged follow‐up interval and whole‐body assessment including CT of thorax or PET imaging may be beneficial. Since 20% of distant metastasis can achieve a long‐term cure if it can be diagnosed and managed at the localized or oligo‐metastasis status.^2^

Our subgroup analysis showed that patients in the HIR–HR group who received VBT alone had an obvious trend of higher locoregional recurrence than those who received EBRT±VBT (7.73% vs. 2.36%, Gray test *p* = 0.08), the recurrence rate was similar to previous trials reported in which varied between 2% and 7%.^18^ We further added specific indicators such as cN0/pN0, LVSI+/−, and stage Ⅰb grade 3 into sub‐risk group analysis. In the HR group, patients in EBRT±VBT showed the trends toward higher 5‐year OS, LRFS, and DMFS compared to VBT alone, especially in LVSI+, pN0, and Ⅰb grade 3 groups, but all of these differences did not reach statistical significance, which is similar to prior studies.^5^, ^30^ Several studies have demonstrated that EBRT can reduce locoregional recurrence but the increased local control did not translate into a survival benefit.^18^, ^31^ Thus, VBT alone is currently recommended for IR or HIR group of patients. However, the optimal adjuvant treatment for the HR group of patients remains controversial.^29^, ^32^ We did not identify a subgroup of patients who would significantly benefit from EBRT, partly due to the high heterogeneity and relatively low proportion of patients in the HR group, indicating the importance of the addition of molecular indicators to further refine the stratification. In addition, our results revealed recurrence profiles in different radiotherapy modes from the perspective of recurrence dynamics. In the HIR–HR group, patients who received VBT revealed a higher hazard of locoregional recurrence and a shape of “double peak” compared to patients who received EBRT, suggesting that EBRT reduced local recurrence (Figure S1). It is noteworthy that the VBT group exhibited delayed relapse behaviors, possibly due to micrometastasis in the unirradiated pelvic lymphatic drainage area. Based on this, it may be beneficial to extend the follow‐up appropriately for this group of patients.

The limitation of this study is its retrospective observational nature. Thus, these results should be interpreted prudently. Metzger‐Filho et al. found that the time‐dependence of an event should be observed over a longer follow‐up in case the effect is overlooked.^33^ Their study performed a relatively long‐term retrospective study, but the changes in indications for adjuvant treatment regimens and approaches during the long‐term course should also be noted. We classified the failure pattern into roughly three types, which is consistent with most previous relevant studies and did not further classify according to the specific site and analyze the relationship between the organ‐specific and risk classification since the number of overall recurrence events was relatively small.^9^, ^10^ Future studies should focus on site‐specific recurrence patterns from the perspective of molecular classification.

## 5 CONCLUSIONS

The present study demonstrated that the patterns of the initial failure recurrence differed widely between risk groups and radiotherapy modalities over time, reinforcing the need for a refined stratification and adjustment of surveillance schemes.

## CONFLICT OF INTEREST

The authors have no potential conflict of interest to report.

## ETHICS STATEMENT

The research protocol was approved by the Institutional Research Ethics Committee of Peking Union Medical College Hospital, Chinese Academy of Medical Sciences & Peking Union Medical College (approval no. S‐K139).

## Supporting information

Fig S1

Supplementary Material

## Data Availability

The data that support the findings of this study are available from the corresponding author upon reasonable request.
