# Supplementary figures and images for: Recurrent patterns after postoperative radiotherapy for early stage endometrial cancer: A competing risk analysis model
Source: Cancer Med. 2021 Nov 15;11(1):257–67. doi: 10.1002/cam4.4423 (PMC8704144; doi:10.1002/cam4.4423)

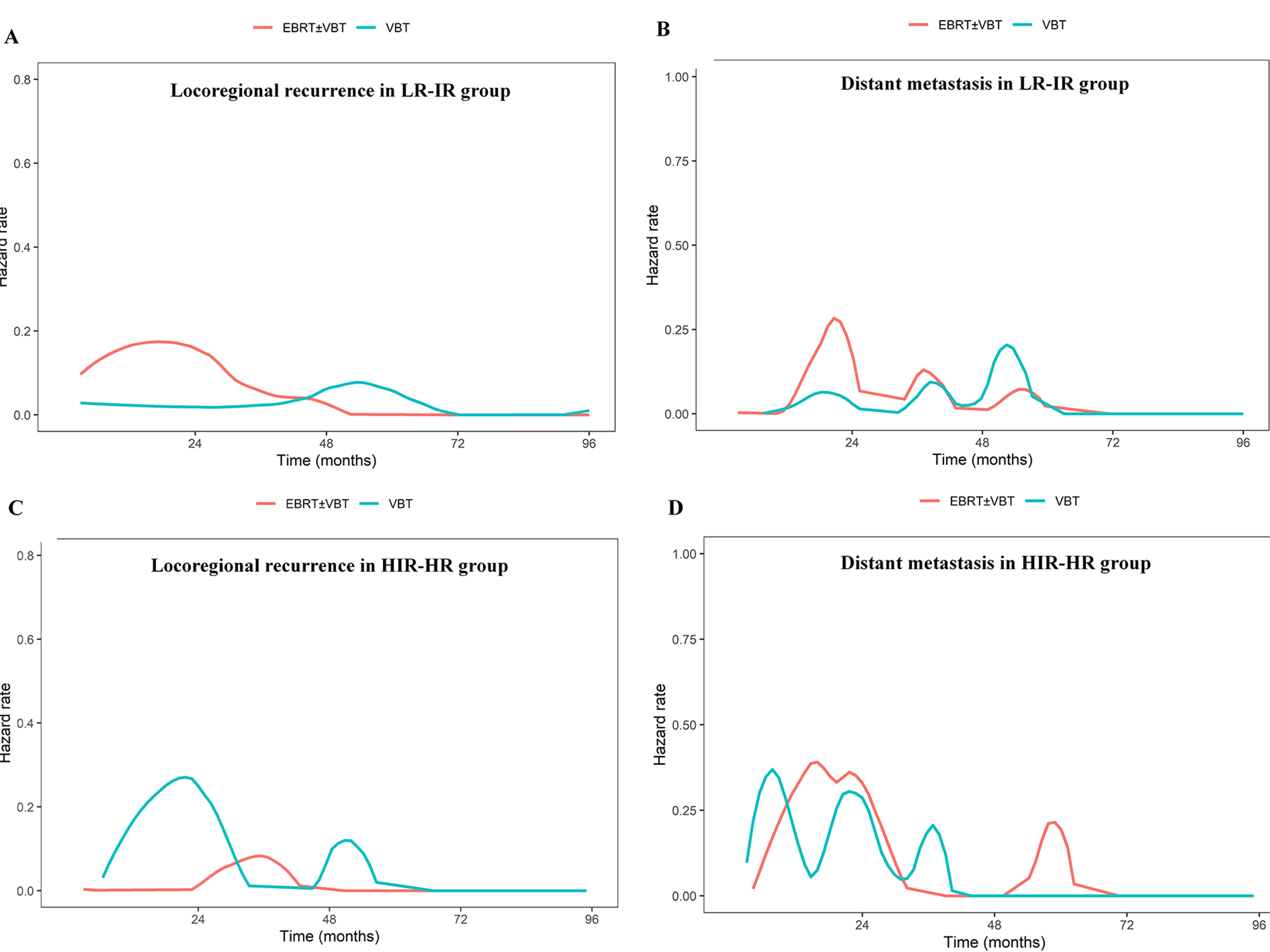

Supplement: Supplementary file 1 — Fig S1 [file CAM4-11-257-s002.tif]
