# Supplementary material for: Recurrent patterns after postoperative radiotherapy for early stage endometrial cancer: A competing risk analysis model
Source: Cancer Med. 2021 Nov 15;11(1):257–67. doi: 10.1002/cam4.4423 (PMC8704144; doi:10.1002/cam4.4423)
Supplement: Supplementary file 2 — Supplementary Material [file CAM4-11-257-s001.docx]

Figure S1. Subgroup analysis of hazard function plots representing different radiotherapy modalities according to the failure patterns over follow-up time: (A) locoregional recurrence in the LR–IR group; (B) distant metastasis in the LR–IR group; (C) locoregional recurrence in the HIR–HR group; and (D) distant metastasis in the HIR–HR group.


**Supplementary Table 1.** **Competing risks regression analysis for hazard rates of recurrence according to risk groups.**

| Site of first recurrence | LR（n=301) | IR  (n=250) | | HIR  (n=164) | | HR  (n=143) | | |  |
| --- | --- | --- | --- | --- | --- | --- | --- | --- | --- |
|  | HR | HR | *P** | HR | *P** | HR | *P** |  |  |
| Vaginal | 1 | 1.200(0.175,8.220) | 0.85 | 1.900 (0.260, 13.930) | 0.53 | 1.160(0.106,12.730) | | 0.9 | |
| Regional† | 1 | 1.285 (0.318, 5.19) | 0.72 | 1.739 (0.433, 6.99) | 0.44 | 2.455(0.537, 11.23) | | 0.25 | |
| Distance† | 1 | 3.780 (1.033, 13.84) | 0.044 | 6.02 (1.672, 21.67) | 0.006 | 12.34(3.454, 24.11) | | <0.001 | |

*Wald test

†Adjusted for age and adjuvant chemotherapy status

**Supplementary Table 2.** **Competing risks regression analysis for hazard rates of recurrence according to the radiotherapy modality in sub-risk groups.**

|  | Site of first recurrence | EBRT±VBT | VBT | *P** |
| --- | --- | --- | --- | --- |
| LR–IR | Locoregional | 1 | 0.72 (0.20, 2.59) | 0.61 |
|  | Distance | 1 | 0.68 (0.22, 2.13) | 0.51 |
| HIR–HR | Locoregional | 1 | 3.06 (0.93, 11.30) | 0.08 |
|  | Distance | 1 | 0.67 (0.32, 1.43) | 0.30 |

*Wald test

**Supplementary Table 3. Subgroup analysis of 5-year OS, 5-year LRFS, and 5-year DMFS**

|  | **5-year OS** | | | **5-year LRFS** | | | **5-year DMFS** | | |
| --- | --- | --- | --- | --- | --- | --- | --- | --- | --- |
|  | **VBT** | **EBRT±VBT** | ***P*-value** | **VBT** | **EBRT±VBT** | ***P*-value** | **VBT** | **EBRT±VBT** | ***P*-value** |
| **LR–IR** | 96.70% | 92.90% | 0.066 | 93.90% | 90.50% | 0.293 | 93.90% | 90.50% | 0.293 |
| **HIR–HR** | 93.20% | 92.60% | 0.999 | 88.60% | 91.60% | 0.28 | 86.70% | 85.70% | 0.903 |
| **LR（n= 301)** | 99.50% | 91.20% | 0.045 | 95.40% | 89.80% | 0.075 | 94.80% | 91.40% | 0.085 |
| pN0(201) | 97.80% | 97.20% | 0.91 | 95.30% | 93.80% | 0.479 | 95.50% | 89.10% | 0.242 |
| cN0(100) | 97.20% | 88.40% | 0.139 | 95.70% | 85.50% | 0.12 | 93.40% | 88.60% | 0.263 |
| **IR（n=250）** |  |  |  |  |  |  |  |  |  |
| pN0(156) | 96.10% | 91.50% | 0.154 | 96.10% | 89.10% | 0.434 | 96% | 86.50% | 0.807 |
| cN0(96) | 89.10% | 97.1%% | 0.239 | 77.80% | 97.40% | 0.007 | 78.60% | 94.40% | 0.027 |
| **HIR (n= 164)** |  |  |  |  |  |  |  |  |  |
| pN0(134) | 96.90% | 94.10% | 0.444 | 91.90% | 91.10% | 0.297 | 92.40% | 90.80% | 0.974 |
| cN0(30) | 87.80% | 88.90% | 0.608 | 72.70% | 91.70% | 0.117 | 74.20% | 88.90% | 0.466 |
| **HR (n = 143)** | 88.30% | 93% | 0.517 |  |  |  |  |  |  |
| pN0(101) | 85.60% | 96.60% | 0.085 | 88.30% | 91.70% | 0.480 | 79.50% | 88.30% | 0.458 |
| cN0(38) | 100% | 82.00% | 0.276 | 80% | 82.80% | 0.894 | 64.30% | 68.80% | 0.809 |
| IB G3 | 80.50% | 90.40% | 0.575 | 78.80% | 87.40% | 0.575 | 74.10% | 84.20% | 0.524 |
| II | 100% | 92.20% | 0.467 | 100% | 90% | 0.400 | 64.30% | 75.80% | 0.697 |
| LVSI+ | 78.10% | 90.20% | 0.492 | 75.10% | 90.50% | 0.295 | 69.60% | 78.90% | 0.488 |
| LVSI- | 100% | 93.20% | 0.257 | 100% | 88.20% | 0.135 | 84.20% | 85.40% | 0.801 |
| Type II | 100% | 92.70% | 0.316 | 100% | 88.30% | 0.248 | 100% | 92.80% | 0.320 |
| Abbreviations: LRFS, local-regional failure-free survival was calculated from the date of surgery to the date of vaginal stump recurrence or regional lymphatic drainage area failure or death due to any cause. DMFS, distant metastasis failure-free survival was calculated from the date of surgery to the date of distant metastasis failure or death due to any cause or the last follow-up time. | | | | | | | | | |

**Supplementary Table 4. Previous studies regarding the recurrence rate and patterns according to risk classification**

| **Author** | **N** | **Stage** | **RT** | **Recurrence rate** | **Recurrence pattern** | **Risk classification** | | |
| --- | --- | --- | --- | --- | --- | --- | --- | --- |
|  |  |  |  |  |  | **Relapse rate** | **5-year cumulative recurrence rate by sites** | **OS** |
| Samual R. et al. (2019) [1] | 2691 | I–II | 25%; | 7.2% | **Locoregional:51%**  Distant:49% | NR | NR | NR |
| E. Vizza et al. (2020) [2] | 697 | I–IIIC2  (I–II:93%) | 71.6% | 19.5% | Locoregional: 36%;  **Distant:64%** | LR:9.6%,  IR:16.7%; HIR:17.1%, HR:40.3% | NR | LR:84.5%, IR:74.1%, HIR:79.3%%, HR 51.2 % (10-year) |
| Tanja et al. (2018) [3] | 2392 | I–IV  （I–II:89%） | 56.6% | 11.6% | Locoregional:36.8%  **Distant:63.2%** | LR:6%;  IR:16%  HR:20.7% | **Locoregional:**  LR,3.6%, IR:5.1%, HR:6.1% **Distant:**  LR:3%, IR:8.6%,7.6% | LR:94.1%, IR:84.5%,  HR:79.3%(10-year) |
| Bendifallah et al. (2017) [4] | 829 | I–III  (I–II:77%) | 51% | 21% | **Locoregional:58.5%**  （LR:72.7%,IR:62.5%.HIR:66.7%.HR:54.4%）  Distant:41.5%  （LR:27.3%,IR:37.5%.HIR:33.3%.HR:45.6%） | LR:9%;  IR:9%;  HIR:16%;  HR:35% | **Locoregional:**  LR:6.5%, IR:6.6%, HIR:16.6%, HR:24.3% **Distant:**  LR:3.3%, IR:3.5%  HIR:5.6%, HR:20.7% | LR:89.0% IR:91.7% HIR:83.2% HR:67.9% (5-year) |
| Carien L. et al. (2003) [5] | 715 | I | 49.5% | 15.4% | **Locoregional:53.6%**  Distant:46.4% | NR | NR | NR |
| Our study | 858 | I–II | 100% | 7.9% | **Locoregional:35.3%**  (LR:46.2%,IR:37.5%,HIR:37.5%,HR:26.1%)  **Distant: 64.7%**  (LR:53.8%, IR:62.50%, HIR:62.50%, HR:73.90% ) | LR:4%;  IR:6.4%;  HIR: 9.8%; HR:16.1% | **Locoregional:**  LR:2.39%, IR:2.57%,  HIR:4.14%, HR:5.97% **Distant:**  LR:1.26%, IR:5.27%,  HIR:7.71%, HR:14.8% | LR:96.1% IR:95% HIR:93% HR: 89.7% (5-year) |
| Abbreviations: N, number of patients. LR, low risk. IR, intermediate risk. HIR, high–intermediate risk. HR, high risk. RT, radiotherapy; CT, chemotherapy, NR, not reported | | | | | | | | |
|  | | | | | | | | |

**References**

1. Francis, S.R., et al., Recurrent early stage endometrial cancer: Patterns of recurrence and results of salvage therapy. Gynecologic oncology, 2019. **154**(1): p. 38-44.

2. Vizza, E., et al., Pattern of recurrence in patients with endometrial cancer: A retrospective study. European journal of surgical oncology : the journal of the European Society of Surgical Oncology and the British Association of Surgical Oncology, 2020.

3. Ignatov, T., et al., Endometrial cancer subtypes are associated with different patterns of recurrence. Journal of cancer research and clinical oncology, 2018. **144**(10): p. 2011-2017.

4. Bendifallah, S., et al., Patterns of recurrence and outcomes in surgically treated women with endometrial cancer according to ESMO-ESGO-ESTRO Consensus Conference risk groups: Results from the FRANCOGYN study Group. Gynecologic oncology, 2017. **144**(1): p. 107-112.

5. Creutzberg, C.L., et al., Survival after relapse in patients with endometrial cancer: results from a randomized trial. Gynecologic oncology, 2003. **89**(2): p. 201-209.
